# Exploiting the diversity of tomato: the development of a phenotypically and genetically detailed germplasm collection

**DOI:** 10.1038/s41438-020-0291-7

**Published:** 2020-05-01

**Authors:** Estefanía Mata-Nicolás, Javier Montero-Pau, Esther Gimeno-Paez, Víctor Garcia-Carpintero, Peio Ziarsolo, Naama Menda, Lukas A. Mueller, José Blanca, Joaquín Cañizares, Esther van der Knaap, María José Díez

**Affiliations:** 1 Instituto Universitario de Conservación y Mejora de la Agrodiversidad Valenciana. COMAV. Universitat Politècnica de València, Valencia, Spain; 2 Department of Biochemistry and Molecular Biology, Universitat de València, Valencia, Spain; 3 Boyce Thompson Institute, Ithaca, NY USA; 4 Institute of Plant Breeding, Genetics and Genomics, University of Georgia, Georgia, GA USA; 5 Department of Horticulture, University of Georgia, Georgia, GA USA

**Keywords:** Agricultural genetics, Plant breeding

## Abstract

A collection of 163 accessions, including *Solanum pimpinellifolium*, *Solanum lycopersicum* var. *cerasiforme* and *Solanum lycopersicum* var. *lycopersicum*, was selected to represent the genetic and morphological variability of tomato at its centers of origin and domestication: Andean regions of Peru and Ecuador and Mesoamerica. The collection is enriched with *S. lycopersicum* var. *cerasiforme* from the Amazonian region that has not been analyzed previously nor used extensively. The collection has been morphologically characterized showing diversity for fruit, flower and vegetative traits. Their genomes were sequenced in the Varitome project and are publicly available (solgenomics.net/projects/varitome). The identified SNPs have been annotated with respect to their impact and a total number of 37,974 out of 19,364,146 SNPs have been described as high impact by the SnpEeff analysis. GWAS has shown associations for different traits, demonstrating the potential of this collection for this kind of analysis. We have not only identified known QTLs and genes, but also new regions associated with traits such as fruit color, number of flowers per inflorescence or inflorescence architecture. To speed up and facilitate the use of this information, F2 populations were constructed by crossing the whole collection with three different parents. This F2 collection is useful for testing SNPs identified by GWAs, selection sweeps or any other candidate gene. All data is available on Solanaceae Genomics Network and the accession and F2 seeds are freely available at COMAV and at TGRC genebanks. All these resources together make this collection a good candidate for genetic studies.

## Introduction

Tomato, *Solanum lycopersicum* var. *lycopersicum* L. (SLL), is one of the most consumed vegetables all over the world with a production that exceeds 180 million tonnes (FAO, 2017). Its cultivation has become highly efficient thanks to the introduction of technological advances and the development of modern varieties. These modern varieties are the result of intensive plant breeding programs since the beginning of the 20th century, and the natural biodiversity of tomato wild species has been key in this success.

The cultivated tomato and its wild relatives came from the Peruvian and Ecuadorian regions of South America. According to allozyme variation, Rick and Fobes^1^ proposed that SLL evolved from *S. lycopersicum* var. *cerasiforme* (Dunal) Spooner, G.J. Anderson & R.K. Jansen (SLC). Recently, Blanca et al.^2,3^ proposed a two-step domestication process from SLC to SLL based on molecular and morphological evidence. The first step involves the pre-domestication of SLC in the Amazonian region of Southern Ecuador and Northern Peru. Subsequently, SLC would have migrated to Mesoamerica where it would be domesticated to SLL. Razifard et al.^4^ proposed that many traits considered typical of cultivated tomatoes arose in South America. However, these domestication traits were lost or diminished once these partially domesticated forms spread to Mesoamerica, where it was finally morphed into the SLL^5,6^. This domestication and diffusion process was accompanied by a selection of alleles related to fruit color, size and shape and also changes in plant architecture^7–10^. This process also included various genetic bottlenecks that progressively narrowed the genetic diversity of modern tomato, compared to its wild species^3,11^. The main loss of variability occurred during the migration to Mesoamerica from the Peruvian and Ecuadorian Amazon region. Most of the allelic variants present in european vintage tomato are already present in these Amazonian SLC populations^3^.

*Solanum pimpinellifolium* L. (SP) is the closest wild relative to SLC and SLL. It is also a red-fruited species and native to coastal areas from Ecuador to Southern Peru. According to its distribution, this species presents varying degrees of genetic variation^12–15^ and morphological differences such as flower and inflorescence size, style exertion, or fruit color^12^. This fact and its capacity to hybridize with tomato, make this species a valuable source of desired traits in tomato breeding. For instance, SP has been used as a genetic source for quality improvement related to solid content, firmness, fruit color^16,17^, volatile compounds^18,19^, or resistance against fungi or viruses such as Tomato leaf curl virus^20^, *Alternaria solani*, *Fusarium oxysporum*, and *Phytophthora infestans*^21^ or *Cladosporium fulvum*^22^. SLC has a worldwide distribution in tropical regions, but it is native to the Andean region of Ecuador and North of Peru^1^. This species is found over a vast range of environmental conditions such as tropical or arid regions, sea level or high altitudes^23^, and it has also been collected at native markets^24^. It usually bears red and small fruits, but Rick and Holle^25^ described a remarkable morphological variability in fruits, plant habit, or leaf size and shape. A higher genetic variability has been described in Ecuadorian and Peruvian accessions^1,2^ due to the development of morphological diversity during a pre-domestication phase. In fact, tomatoes collected in local markets of Ecuador were morphologically classified as vintage tomato; but they have been genetically classified as SLC^3^. These studies and data show that SLC from Northern Peru are very close to Mexican and vintage tomatoes. Despite that, Amazonian SLC has not been used frequently for tomato improvement as opposed to SP. Furthermore, SLC has been characterized as a valuable genetic source for abiotic and biotic stresses, such as moisture-tolerance^26^ or resistance to root rot caused by *Phytophthora*^27^; traits related with the global climate change and sustainability challenges currently facing agriculture.

Most modern breeding programs have usually focused on resistance, yield and quality traits, such as firmness, color, or texture^28^, plant habit and adaptation to machine harvesting in processing cultivars or traits related to fruit appearance for fresh market^28^. However, nowadays, the new objectives of tomato breeding focus on sustainable production or adaptation to unfavorable environmental conditions due to climate change and nutritional quality. The genetic variation of exotic germplasm collections has been used in tomato breeding to bypass the limited genetic diversity of SLL. These germplasm collections have mainly included *S. pimpinellifolium, S. chilense* (Dunal) Reiche*, S. peruvianum* L. s. str.*, S. habrochaites* S. Knapp & D.M. Spooner and *S. pennellii* Correl. Thus, the maintenance and characterization of germplasm collections are essential in order to achieve these breeding goals. Germplasm is a good source of natural allelic variants, useful for genetic analyses and subsequent breeding applications. Consequently, the creation of genebank collections characterized at genetic and phenotypic level is a primary objective for a sustainable breeding. In addition, it is crucial that these data and genetic resources are easily available to the scientific community to exploit this extensive amount of information.

The advent of NGS technologies has created a huge amount of available genetic information about germplasm held in genebanks^29^ that can be useful for improving breeding cultivars^30^. For instance, the availability of its genomes in association with its characterization at phenotypic and molecular level allows the development of genome-wide association studies (GWAS). GWAS studies have already identified regions of the genome related to morphological and metabolic diversity^31,32^. For example, Bauchet et al.^31,33^ detected associations for traits such as fruit weight, flowering time, early fruit development, malate, and phenalyacetaldehyde/phenylethanol content. Finally, the first meta-analysis of GWAS has revealed numerous candidate genes involved in tomato flavor^34^. Full genome sequences have been published in several studies and more than 725 genome sequences of tomato accessions are available^35–39^. A pan-genome analysis of tomato including SLL, SLC, and SP has discovered 4873 genes that are not present in the reference genome^37^ thus increasing the interest of these populations for tomato breeding. Once a candidate region of the genome, gene or SNP has been characterized as significantly associated with a trait, it is necessary to validate its role in the control of the trait by using segregating families or mutants. However, this latter step sometimes becomes limiting as the development of such populations is time consuming and costly.

In the present study, we have morphologically characterized the variability of fruit, flower, and vegetative characters from a collection of 163 tomato accessions of the Varitome project, for which the full genome is available^37^. These accessions include SP, SLC, and SLL and represent the diversity at the center of origin and domestication of tomato. We have annotated the identified SNPs within our collection using SnpEff. We have performed GWAS analysis for all of our morphological descriptors with the aim of detecting candidate regions. In addition, a collection of segregating families has been developed by crossing the complete set of accessions with a representative accession for each of the three species. These populations could help to speed up the validation of candidate genes and SNPs. The combination of passport, phenotypic, genetic information, and germplasm with easy accessibility converts this collection into a powerful instrument for genetic studies and breeding.

## Results

### Morphological analysis

**Fig. 1:**
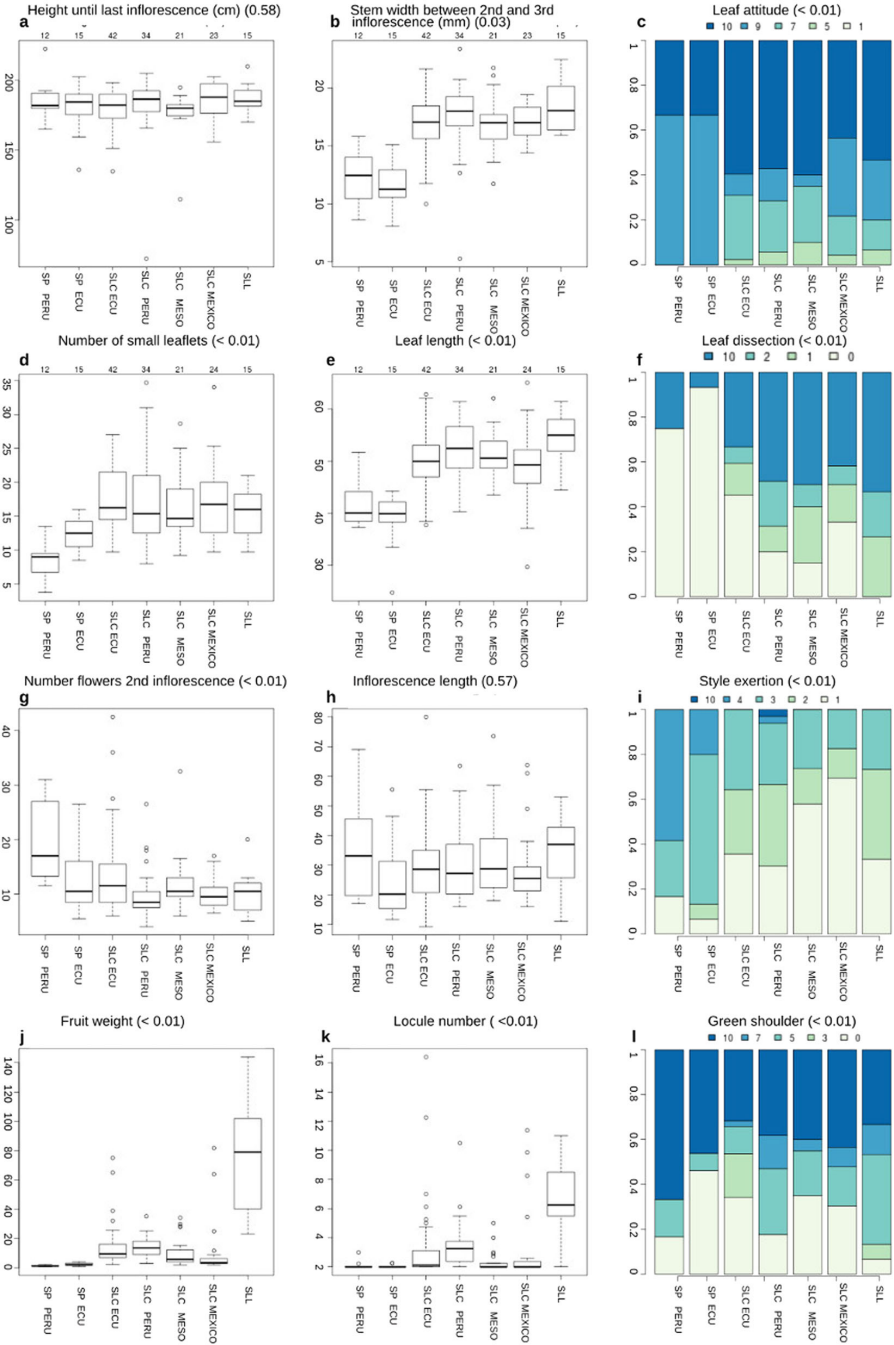
Morphological variation. Distribution for eight quantitative and four qualitative morphological traits related to vegetative (**a**–**c**), leaf (**d**–**f**), flower (**g**–**i**), and fruit (**j**–**l**) descriptors for each geographical group. *p* Values (in brackets) of the differences between species are shown. Morphological traits were measured as follows: **a** Plant height until last inflorescence, measured in cm. **b** Stem width between second and third inflorescence, measured in mm. **c** The way that leaves are held naturally (1: semi-erect, 3: semi-horizontal, 5: horizontal, 7: horizontal-drooping, 9: drooping, 10: accessions that exhibited variability for their measures). **d** Number of small leaflets. **e** Leaf length, measured in cm. **f** Leave dissection (0: low, 1: intermediate, 2: high, 10: accessions that exhibited variability for their measures). **g** Number of flowers in the second inflorescence. **h** Distance from the stem to the last flower of the inflorescence. **i** Position of the style in relation to stamens (1: inserted, 2: same level as stamen, 3: slightly exerted, 4: highly exerted, 10: accessions that exhibited variability for their measures). **j** Fruit weight, measured in grams. **k** Number of locules in the transversal section of the fruit. **l** Presence and color of green shoulder (0: uniform, 3: light green, 5: medium green, 7: dark green, 10: accessions that exhibited variability for their measures)

**Fig. 2:**
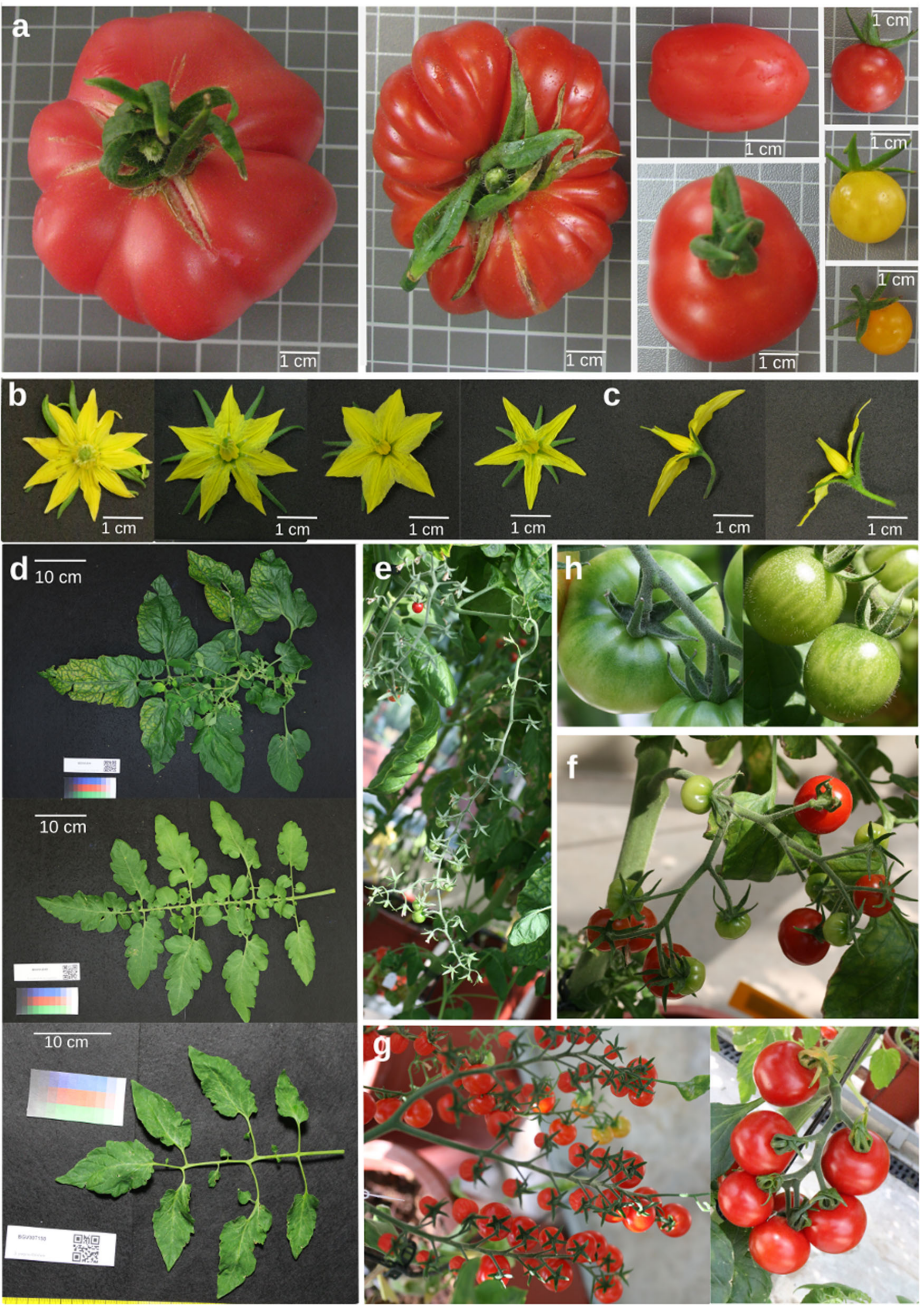
Diversity in leaf, fruit, flower, and inflorescence traits. **a** Tomato fruit size, shape, and color. **b** Variability for flower complexity, related to the number of petals and sepals and their sizes. **c** Differences between exerted and inserted styles. **d** Diversity in leaf size, number of small leaflets and border or dissection of small leaflets. **e** Uniparous inflorescence. **f** Forked inflorescence. **g** Irregular inflorescence. **h** Differences between presence and absent of green shoulder

A germplasm collection of 163 accessions was selected with the aim of representing the geographical, morphological, and genetic diversity of tomato and its closest wild relatives at their region of origin (Supplemental Fig. S1 and Supplemental Table S1). These materials consisted of 15 accessions of SLL from Mexico; 121 accessions of SLC coming from Ecuador, Peru, Mexico, and different countries of Mesoamerica and 27 accessions of SP from Ecuador and Peru. The accessions have been grouped based on their geographical origin and on previous genetic studies^2,3^. Plants were evaluated for a total of 54 morphological traits (Supplemental Table S2) describing the variability of this collection for plant architecture, leaves, inflorescences, flowers, and fruits (Figs. 1, 2, Supplementary Table S3). The lowest morphological variability was found in quantitative traits related to plant architecture such as height until first or last inflorescence (Fig. 1a) or stem width (Fig. 1b). However, SP can be differentiated from the rest of species by this last trait. Qualitative traits related to plant architecture showed that most accessions had an indeterminate growth habit and that a wide range of variation related to the way that leaves were held naturally exists (Fig. 1c).

Quantitative traits related to leaves were the leaf size, the number of primary leaflets and small leaflets; whereas the qualitative ones described the leaf morphology, complexity and leaflet dissection, and shape. The collection exhibited a low variability for number of primary leaflets, while differences were considerably greater for leaf size and the number of small leaflets, as it is shown in Fig. 2d. Figure 1d, e show that SP group was characterized by smaller leaves whereas the maximum values were found in SLC group. Observations related to the type of leaf revealed that SP group was generally characterized by pimpinellifolium type leaf, SLC group exhibited all types but generally leaves were classified as standard ones and SLL exhibited standard and double feathered types. SP leaf was generally characterized by a lack of dissection (Fig. 1f) and entire or undulating borders. However, SLC and SLL groups exhibited more variability in leaflet dissection (Fig. 1f) and border.

Traits related to inflorescences included inflorescence length, number of flowers per inflorescence or type of inflorescence, whereas flowers were evaluated for number of petals and sepals and their length, width, and style exertion, among others. The values observed for inflorescence length and the number of flowers per inflorescence demonstrated a wide variability (Fig. 1g, h). In addition, the complexity of the inflorescence exhibited a considerably diversity (Fig. 2e–g). For flower traits, most accessions had between 5 and 6 petals and sepals per flower but several accessions were much more complex (Fig. 2b). SP Ecuador and Peru and SLC Mesoamerica exhibited the simplest flowers and low variability, as oppossed to the complexity observed in SLC Ecuador, SLC Peru, SLC Mexico, and SLL. Finally, the observed variability related to the position of style is represented in Figs. 2c and 1i.

The high variability for fruit weight and locule number is shown in Fig. 1j, i, respectively. SP was characterized by the smallest fruits whereas SLL group presented the biggest. However, the highest variability appeared in SLC group which produced smaller values than SP or bigger than SLL. Finally, qualitative traits related to fruit appearance revealed that most accessions produced red fruits, although other colors were also present. Some accessions belonging to SP species presented an intense red fruit, and others belonging to SP Peru and SLC Mexico groups exhibited colors ranging from yellow to orange. Other qualitative fruit traits presented high variability, such as the presence and intensity of green shoulders (Figs. 1l and 2h). This variability in fruit size, color and shape is shown in Fig. 2a.

### Genome-wide association analysis

**Fig. 3:**
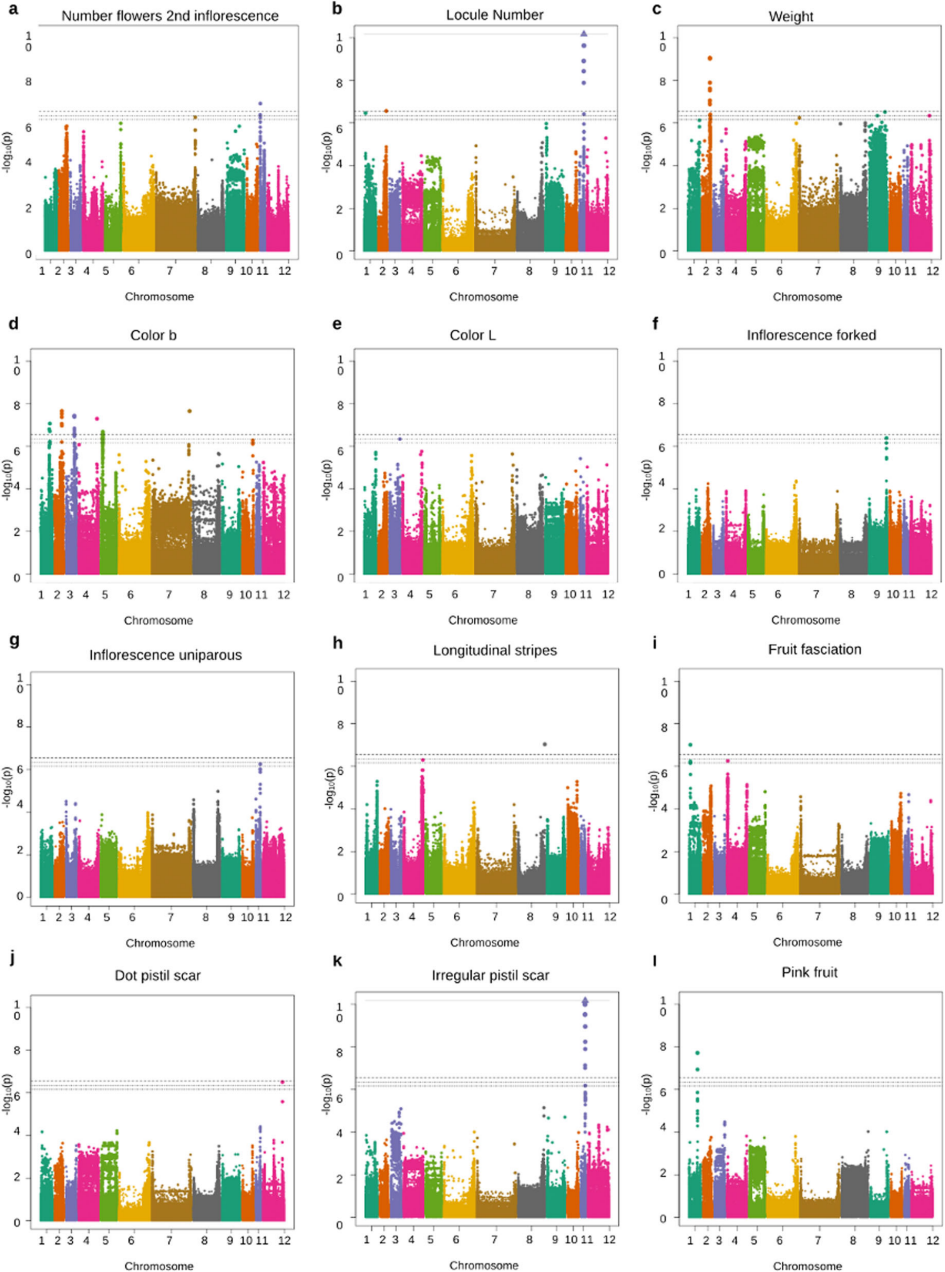
Genome-wide association results for some traits that showed significant association

GWAS analysis revealed significant associations with a total of 15 traits. We found SNPs associated with eight quantitative traits (Fig. 3 and in Table S5). For the total number of inflorescences and petal length traits, each was associated with a single SNP located on chromosomes 1 and 9, respectively (Table 1). The number of flowers in the second inflorescence revealed associations with two SNPs located in chromosome 7 and 11. The result of leaf length analysis revealed two associated regions on chromosome 2 and 8. Associations with locule number were detected on chromosome 1, 2, and 11 and associations with fruit weight were detected on chromosomes 2, 7, 9, and 12. The most remarkable associations occurred on chromosome 2, since associated SNP were located in the genomic region where *locule number* and *fw2.2* QTLs have been described. On chromosome 11, the association with the trait number of locules is located on the *fas* gene. On chromosome 9, the previous QTL *fw9.2* was detected for fruit weight. Interestingly, there is not a close described QTL for chromosomes 1 and 12 related to locule number or weight, respectively. Several associations for fruit color have been detected, listed in Table 1 and Table S5. For instance, GWAS for LAB color space’s *b* value revealed associations on chromosome 1 that were located in a genomic region with an annotated gene as carotenoid cleavage dioxygenase 1B. Regions on chromosome 3 and 10 were close to annotated genes involved in yellow and orange fruit flesh. Finally, the analysis detected also a region on chromosome 5 that has not been previously described for this trait. For LAB color space’s *L* value, the association detected on chromosome 3 lacks of annotated genes. GWAS analysis also showed associations between SNPs and qualitative traits, as it is shown in Fig. 3 and Table 1. The genomic region on chromosome 9 associated to dark-green leaves lacked annotated genes and only one significant SNP for low petal curvature was detected on chromosome 7. For the type of inflorescences, one genomic region on chromosome 9 could be involved in forked inflorescence and chromosome 11 could carry another region that could be involved in uniparous inflorescence. For fruit traits, associations with the presence of longitudinal stripes, fasciated fruit, ribbing at calix end, and fruit scar were detected. The most remarkable result was the association for irregular pistil scar, covering a region of 355 kb on chromosome 11 that included several genes and three of them were also associated to ribbing at calix end. Finally, two genomic regions on chromosome 1 were associated with pink fruits (175 kb) and fasciated fruits (200 kb).

**Table 1:** Summary of significant associations detected for quantitative and qualitative traits. For each trait, the position in bp on the chromosome, the corresponding annotated gene or close annotated gene, known genes related to the trait and number of SNPs with a high putative effect are shown

Trait	Chromosome	Position	Locus name	Close annotated locus name	Know QTL	Number of high impact SNPs
Color b	1	78,911,282–82,203,699	*Solyc01g079760, Solyc01g087260*			Solyc01g087260: 2
Color b	2	44,794,084	*Solyc02g080610*			
Color b	3	52,153,309–52,406,077	*Solyc03g081260, Solyc03g081300, Solyc03g082480*			Solyc03g081260: 14
Color b	5	9,596,743–9,779,918	*Solyc05g015030, Solyc05g015070*			Solyc05g015030: 2
Color b	10	60,308,826	*Solyc10g078510*			
Color L	3	65,751,759				
Dark-green leaf	9	65,565,848–65,566,230	*–*	*Solyc09g072880*		
Fruit fasciation	1	3,690,368–3,918,681	*Solyc01g009510*			Solyc01g009510: 1
Fruit fasciation	4	3,935,728	*–*			
Fruit weight	2	49,587,330–52,662,216	*Solyc02g087050, Solyc02g087780, Solyc02g087810, Solyc02g091330*		*fw2.2*	Solyc02g087050: 1, Solyc02g091330: 1
Fruit weight	7	1,318,982	*Solyc07g006520*			Solyc07g006520: 1
Fruit weight	9	31453421	*–*		*fw9.2*	
Fruit weight	9	60,775,035	*–*		*fw9.2*	
Fruit weight	12	61,805,734	*Solyc12g055810*			
Inflorescence forked	9	68,492,821–68,496,154	*Solyc09g082830*			
Inflorescnce uniparous	11	55,020,323–55,194,697	*Solyc11g071600, Solyc11g071830, Solyc11g071840*			Solyc11g071600: 1
Leaf length	2	39,577,220–39,581,181	*–*	*Solyc02g069760*		
Leaf length	8	60,002,113–60,010,971	*–*			
Locule number	1	3,717,866	*–*			
Locule number	2	49,617,538	***Solyc02g087100***		*locule number (lc)*	Solyc02g087100: 1
Locule number	11	54,838,887–55,194,697	***Solyc11g071310****,* ***Solyc11g071600****,* ***Solyc11g071820****,* ***Solyc11g071830****,* ***Solyc11g071840***		*fas and fw11.3*	
Longitudinal stripes	4	60,283,297	*–*	*Solyc04g074290*		
Longitudinal stripes	8	65,493,583	*Solyc08g082820*			Solyc08g082820: 2
Number flowers in second inflorescence	7	65,679,033	*–*			
Number flowers in second inflorescence	11	101,719–612,480	***Solyc11g005110***	*Solyc11g005510, Solyc11g005570*		
Petal curvature low	7	1,760,949	*–*	*Solyc07g006910*		
Petal length	9	69,065,327	*–*	*Solyc09g083410*		
Pink mature fruit	1	78,736,178–78,911,282	*Solyc01g079760*	Solyc01g079620		Solyc01g079620: 5
Pistil scar dot	12	61,805,734	*Solyc12g055810*			Solyc12g055810: 2
Pistil scar irregular	11	54,838,887–55,194,697	*Solyc11g071310, Solyc11g071580, Solyc11g071600, Solyc11g071820, Solyc11g071830, Solyc11g071840*			Solyc11g071580: 9
Ribbing calix end	11	55,020,323–55,183,870	*Solyc11g071600, Solyc11g071820, Solyc11g071830*			
Total number of inflorescences	1	3,717,866	*–*			

### Annotation and prediction of the SNP effects

The SNPs identified in this collection are available at Solanaceae Genomics Network (https://solgenomics.net/). The SNPs were annotated and their putative impacts were predicted by using SnpEff. The number of effects were classified by the impact of these variants, type of effect, and region. The lowest number of variants was detected in our SLL group. SLC groups had a variation between SLL and SP groups with lower levels of variants in SLC Mexico. A total of 37,974 out of 19,364,146 SNPs detected in this collection have been designated as high impact in the SnpEff analysis. The number of variants per type and the number of effects by impact for each group are summarized in Table 2. However, it is important to take into account that the number of SNPs is influenced by the different number of accessions in each of the groups. Among other mutations, the generation or the loss of stop codon could be one of the most interesting changes because the synthesis of an essential protein could be affected and its function would change. As shown in Fig. S2, the same pattern of this SNP distribution was observed for the number of these mutations. Finally, genomic regions of candidate genes from GWAS analysis were used to find out allelic variants in the collection labeled as high impact. These 37,974 SNPs with a high putative impact were related to 12 candidate genes (Table 1) and are summarized in Supplemental Table S6.

**Table 2:** Result of the number of variants per type and the number of effect by impact for each geographical group from SnpEff

	Number of variants per type			Number of effects by impact			
	SNP	INS	MIXED	HIGH	LOW	MODERATE	MODIFIER
All samples	15,700,927	2,736,310	926,909	45,111	143,153	196,099	27,035,721
SP Ecuador	7,705,076	1,976,689	738,373	28,070	77,621	97,472	17,935,822
SP Peru	7,752,552	1,995,127	747,838	28,673	81,778	102,291	15,074,861
SLC Peru	6,476,228	1,834,571	699,351	25,197	65,290	83,083	12,905,503
SLC Ecuador	6,358,145	1,726,621	615,355	24,137	63,308	81,036	12,515,017
SLC Mesoamerica	5,963,635	1,628,278	619,202	21,682	55,103	72,443	11,580,385
SLC Mexico	3,781,905	1,112,989	371,858	13,919	31,684	45,342	7,342,190
SLL	658,721	387,752	87,763	4178	9168	11,567	1,793,184

*SNP* single-nucleotide polymorphism, *INS* insertion, *MIXED* multiple-nucleotide and InDel

### Development of segregating families

The whole collection was crossed with BGV007109 (SP), LA2278 (SLC), and Money Maker (SLL). The 163 accessions were used as female parents to obtain the F1 generations, except for some accessions, mainly SP, where flowers were too difficult to emasculate due to their small size. F1 plants were self pollinated to obtain the F2 generations. A collection of 485 F1 populations and 457 F2 population were achieved (Supplemental Table S4). Considering that most of the cross collections from each accession can have various independent F2 populations, created from different F1s, the total number of different F1 and F2 populations are 1430 and 672, respectively. The seeds of these segregating families are available at COMAV.

## Discussion

### Morphological variability

The results of our study revealed a wide range of diversity in our collection for most of the evaluated traits, mainly related to leaves, fruit shape and size, and color or flower morphology. Regarding SLC group, it generally exhibited the highest grade of morphological diversity since the group comprises a wide range of geographical origins. For traits related to leaf shape and size, this group displayed much higher diversity in comparison to the simpler leaves of SP^24,25^.

The high variation related to fruit color and shape was an interesting source of variation for breeding and genetic purposes. Some SP and SLC accessions, collected in Peru and Mexico respectively, exhibited colors ranging from yellow to orange. In fact, yellow fruits have been previously described in these sites^12,24,25^. The fruit shape of SLC group exhibited a considerable variation ranging from round to flattened, fasciated, or elongated fruits. The transition from small and uniform fruits of SP to diversity in fruit size, shape, and locule number was a consequence of variations in flower complexity and an increase in ovary size. For example, the appearance of fasciated phenotype (*fas*) has been suggested to have arrived in Europe from Mexico in the 16th century^9^. These changes in fruit size and shape have been described to be a consequence of derived alleles of *fas*, *sun*, *ovate*, and *lc* genes^9^. According to the study of Blanca et al.^3^, some accessions from our SLC group carry *fas* and *ovat*e and some of our SLL also carries derived alleles. These results support our observations and these derived alleles could be an explanation for the diversity that we have detected.

Changes in flower complexity and style exertion could be other interesting changes related to domestication and further selection processes. For example, the number of petals and sepals tended to increase in SLC and SLL groups, although not in SLC Mesoamerica. The style position was also altered from highly exerted in SP Peru to slightly exerted or even inserted in SP Ecuador. In SLC, the degree of style exertion tended to decrease from Amazonian SLC to SLC Mexico, whereas in the SLL group it tended to be inserted. This correlation between stigma exertion and SP geographical origin had been previously described^12,40^, as well as the variation observed in SLC from South America^24,25^. This insertion process is related with the migration from the center of origin and resulted in the increasing of the autogamy levels. Interestingly, two different subgroups can be discerned in SLC Mexico, accessions collected as wild that exhibited inserted styles, and accessions with fruit size similar to cultivated tomato that were characterized as exerted ones. This presence of exertion is also detected in SLL and probably related to fasciated and big sized fruits.

### Genetic variability

As expected, the highest level of diversity was found in Peru and Ecuador for both SP and SLC groups^2,3^. Rick and Fobes^1^ described that variation in SLC depended on its geographical origin, being SLC from other countries less variable than SLC from these regions. The analysis within the SLC group also revealed a considerable decrease in the number of SNP variants detected in SLC Mexico. This is in agreement with the loss of variability that took place during the migration to Mesoamérica^2,3,41^. The detected 37,974 SNPs labeled as high impact show that this collection may be an interesting source of new alleles. This is supported by the SNPs with a high putative effect that were detected for some of the candidate genes from GWAS analysis (Supplementary Table S6). For example, candidate genes related to yellow fruit color had a total of 18 allelic variants with high effect. Two of them correspond to a carotenoid cleavage dioxygenase 1B (*Solyc01g087260)*, 14 of them correspond to a protease-like protein (*Solyc03g081260*), and the last 2 correspond to a homeobox leucine-zipper protein (*Solyc05g015030)*. Also, a high impact SNP was detected in the genomic region where *lc* gene is located. Besides, the genomic region associated to *fw2.2* presented two allelic variants with high effect. These variants are related with a nodulin MtN21 family protein *(Solyc02g087050*) and with an uncharacterized protein (*Solyc02g091330*).

### GWAS analysis

GWAS analysis revealed a total number of 107 SNPs associated to eight quantitative traits and 30 SNPs associated to seven qualitative traits. This analysis has allowed the identification of known and novel genes for these traits. In addition of the QTLs for flowers per inflorescence previously described on chromosomes 2, 3, and 5^42^, our analysis identified a possible novel genomic region on chromosome 11 which carries genes encoding for Agenet and cellulose synthase proteins. Agenet has been described to be involved in flower development^43^ and the expression of cellulose synthase has also been detected in flowers of *Arabidopsis thaliana*^44^. The association identified for forked inflorescence on chromosome 9 corresponds to a SNP in *ARGONAUTE 1* gene (*Solyc09g082830*). This gene is a member of AGO gene family, which is known to regulate vegetative and reproductive development and stress response^45^. The expression of these genes have been detected in flower and fruit of tomato^46^. A significant association with uniparous inflorescence was identified on chromosome 11 and was located approximately 5 Mb away from a mapped region which is considered to be involved in branched inflorescences of *fin* mutants^47^. Six associations for dark green leaves were detected on chromosome 9. An annotated gene as chloroplast FLU-like protein was located 2 kb away from this region. FLU is a nuclear-encoded plastid protein that interacts with enzymes involved in chlorophyll synthesis^48^. Besides that, some new identified SNPs lacked in functional annotation, for example SNP associated to the total number of inflorescences. Finally, another several traits were associated with SNPs located on genes that were not apparently related to the trait they are associated with, such as the association between leaf length and a RING-finger protein-like which could regulate ubiquitination processes^49^ or the association between fruit longitudinal stripes and heat shock proteins. All these novel detected regions would require further experiments for validation and identification of candidate genes suitable for tomato breeding.

As expected, our analysis has identified several SNPs located close to genes or candidate regions previously characterized. GWAS analysis has allowed the identification of previously described *loci* associated to fruit size such as *fw2.2*^50^, *fw9.2*^51^*, locule number (lc)*^9^, and *fas*^9^. For instance, we detected an association between fruit weight and SNPs close to *fw2.2* and also close to SNPs that have already been identified in other GWAS analysis^31,52^. In case of *lc* and *fas* genes, our study revealed associations between the trait number of locules and SNPs located genetically close to both QTLs, which are located on chromosome 2 and 11, respectively. Sacco et al.^52^ detected *lc* gene and also one of our annotated candidate genes on chromosome 11, *Solyc11g071840*. This detected region on chromosome 11 was located in a region previously described as *fas* and really close to the annotated *fw11.3*. The *fw11.3* is a QTL controlled by cell size regulator, which regulates weight by the control of cell size in the pericarp^53^. Strikingly, no association has been found between the qualitative trait fruit fasciation and chromosome 11 (where *fas* gene is located). However, two regions previously undescribed that could be associated to fasciated phenotype on chromosomes 1 and 4 were revealed. Despite that *fas* gene is not located in these detected regions, this result suggests the involvement of new genome regions. In fact, the SNP located on chromosome 1 at 3,717,866 bp was also associated with the number of locules in our analysis.

An association signal for fruit color was identified on chromosome 1 and located in a region with a candidate gene described as carotenoid cleavage dioygenase 1B. Carotenoids are important factors implied in fruit color and modifications or absence of their syntheses are the reason for the orange color of mutants such as tangerine (*t*), delta (*Del*) and beta (*B*) or the yellow flesh (*r*) mutant^54^. The genome region involved in this *r* mutant is located on chromosome 3 at 9 Mbs from our associated region. For the pink color, associations were located in a genomic region with an annotated gene as colorless fruit epidermis *(y* gene). This association between the pink fruit color and this gene had already been detected by GWAS analysis and a deletion in this region has been hypothesized to control this trait^52^.

### The utility of this germplasm collection

The present work has revealed a wide range of variability in our collection. The novelty of our study is the inclusion of a wide range of geographical origins of SLC accessions and SP from North Ecuador, which has not been widely studied. Moreover, the potential of Andean SLC is still not widely explored and it could be a novel source of interesting agronomic traits for tomato breeding. The genetic variability present in SLC from the Amazonian region is huge in comparison with the variability of the traditional tomato, although it has notably increased recently due to introgressions from wild species. The close phylogenetic relatedness of SLC makes this species specially useful for being exploited in tomato breeding, much more than other more phylogenetically distant species.

The high morphological variability found in our study may be an evidence of the potential variability in other traits not evaluated in this work. This collection is being analyzed for biochemical composition of fruit and deeper approaches for specific morphology analyses of fruit in the context of the Varitome project. The genome sequences of all these accessions are published and available, together with the identified annotated SNPs. The number of allelic variants present in this collection is huge and many of them may have interesting effects, such as lost or gained stop codons, frameshift variants or splice variants. A pan-genome analyses that includes the set of accessions we have used in our work, has described 4873 genes not present in the reference genome^38^. Part of this gene variability is present in our collection and easily accessible. This increases its usefulness, and makes our collection in one of the most characterized of tomato and related species.

The GWAS study has shown that the size and population structure of this collection make it feasible for this type of analysis. Further studies with other traits probably will increase the identification of candidate genes and alleles. Seeds from these sequenced accessions are available from two genebanks, one in Europe (COMAV) and the other in America (TGRC). The characterized and sequenced plants came from a double round of self-pollination of a single plant, so they are quite homozygous and it is possible to use the genotype data to do other GWAS analyses with other traits.

Different segregating families have been developed and have led to the creation of a powerful tool to speed up genetic studies based on this collection. The use of three different parents in crosses with all accessions, allows the testing of the same alleles in different genetic backgrounds. The F2 populations will facilitate the analysis of the segregation of any variant in this collection. Studies can be conducted starting from SNP alleles, presence or absence of a determinate gene or phenotypic variants. By choosing an accession carrying a selected allele and one of the three parental accession which carries the alternative allele, the F1 and F2 segregation families are available for the genetic study of this variant. The availability of these segregating families allows to speed up research to confirm possible candidate genes. Besides sparing the effort of developing segregating families, researchers could analyze different natural mutants of the same gene or study the mutation effect in different genetic backgrounds.

The usefulness of this collection is based on the fact that all these resources are freely and easily available. Seeds of the original and self-pollinating accessions and F2 families are available at COMAV and TGRC genebanks. Passport and characterization data, pedigree information, genome sequences, SNPs and GWAs results are available and integrated at Solanaceae Genomics Network (solgenomics.net). All these resources build up a powerful platform for tomato genetics and breeding that could be reinforced with new studies performed on it.

## Material and methods

### Plant material

A germplasm collection of 163 accessions was selected with the aim of representing a broad range of geographical, morphological, and genetic diversity. These plant materials consisted of 15 accessions of SLL from Mexico; 121 accessions of SLC coming from Ecuador, Peru, Mexico, and different countries of Mesoamerica and 27 accessions of *Solanum pimpinelifollium* (SP), from Ecuador and Peru. Accessions were grouped according to their geographical origin and previous genetic results^2,3^, as is shown in Fig. S1 and Table S1. These accessions were provided by different germplasm banks such as Tomato Genetics Resource Center (TGRC), United States Department of Agriculture (USDA), and Instituto Universitario de Conservación y Mejora de la Agrodiversidad Valenciana (COMAV) of Universitat Politècnica de València. Passport data of these accessions are available in Supplementary Table S1 and COMAV, Solanaceae Genomics Network web pages. For each accession, seeds obtained after a double round of self-pollination from a single plant of each original accession were collected and they were used either for the morphological and genetic characterization and for the creation of segregating populations.

### Morphological characterization

Plants used for the morphological characterization were cultivated in a greenhouse at Universitat Politècnica de València (Valencia, Spain) during the spring-summer seasons of 2016. Plants were grown in 12-l pots with coconut fiber and fertirrigated under standard dosages for tomato in our area. A completely randomized experimental design was conducted with two plants per accession, each replicate in a different greenhouse.

Twenty-six quantitative and 27 qualitative traits based on the descriptors developed by IPGRI^55^, mainly related to plant architecture, inflorescences and flowers, leaves and fruit size were evaluated. Some descriptors were modified for a better representation of the morphological variability exhibited in the collection. The descriptors and their definitions are listed in Supplementary Table S2. All traits were added to the Solanacea Phenotype Ontology, available at SGN (https://solgenomics.net/search/traits).

Prior to any analysis, all traits were manually curated to detect possible errors. Differences due to a greenhouse effect were assessed using Student’s *t* test and Mann–Withney-Wilcoxon test for quantitative traits depending on whether the data was normally distributed. Fisher’s exact test was conducted on qualitative data. As no differences between the two greenhouses were found, data from both greenhouses were joined, and the mean value was calculated for quantitative traits. For qualitative data, a new level for each qualitative trait (named as 10) was created to include the accessions which presented different scale values for this qualitative trait. Robust ANOVA and Fisher test were conducted to detect significance differences between species, depending on whether the data was quantitative or qualitative. A Bonferroni correction of *p* values was conducted.

### Genetic analysis

Genome sequences of the accessions of this collection have been published previously^35^ and the SNPs identified in these accessions are publicly available in Solanaceae Genomics Network (https://solgenomics.net/projects/varitome). Using these data, the collection of SNPs has been annotated to detect the localization and possible impact of changes using SnpEff^56^, and statistics were calculated for each geographical group.

GWAS between genotypes and phenotypes were calculated for all quantitative and qualitative traits using R package GENESIS v.2.14.1^57^. A total number of 1,479,141 high quality SNPs were used for the GWAS analysis. A PCoA has been done to visualizate genetic structure (Supplemental Fig. S3). To test the association, a generalized linear mixed model using the genetic relationship matrix (GRM) as random effects was used in order to account for population stratification. GRM was computed using GCTA v.1.92.1^58^. For count data, a Poisson distribution of residuals was assumed, while for the rest of the quantitative data a Gaussian distribution was applied. Normality was checked using a Shapiro–Wilk normality test and a Box–Cox power transformation was used when necessary. For qualitative traits, a binomial distribution was assumed. For multinomial qualitative traits, each category level was treated as a dummy binary variable. Quantile–quantile plots were used to assess the GWAS model (Supplemental Fig. S4). Significant level of association was estimated using GEC (Genetic type 1 Error Calculator) v.0.2^59^.

### Development of breeding population

In order to help to exploit the variability detected in our collection and facilitate its use to the research community, F1 and F2 generations were constructed for the 163 accessions by crossing each accession with one accession representative of each species (SP, SLC, and SLL). The accessions, BGV007109 of SP, LA2278 of SLC and Money Maker of SLL were selected as parents. Each fruit from each individual cross was maintained separately in order to facilitate the detection of possible mistakes. Two different F1 plants of each combination were self pollinated to obtain the set of two independent F2 breeding populations. The culture for the F2 family generation was done during the years 2017–2019 in the Centro de experiencias Cajamar de Paiporta (Valencia, Spain). Plants were grown in greenhouses in soil and fertirrigated under standard dosages for tomato in our area.

A complete list of these available materials is recorded in Supplementary Table S4.

## Supplementary information

Figure S1

Figure S3

Figure S4

Table_S1

Figure S2

Table S2

Table_S6

Table_S3

Table_S4

Table_S5

## Data Availability

Sequences, SNPs, passport data, characterization data, and images of the original collection are available in Solanaceae Genomics Network (https://solgenomics.net/). Seeds of the original germplasm collection are available by request to COMAV genebank (mdiezni@btc.upv.es) and to the TGRC (https://tgrc.ucdavis.edu/). Requests for the available seeds of F1 and F2 families should be addressed to COMAV genebank.
