# Supplementary figures and images for: Exploiting the diversity of tomato: the development of a phenotypically and genetically detailed germplasm collection
Source: Hortic Res. 2020 May 1;7:66. doi: 10.1038/s41438-020-0291-7 (PMC7192925; doi:10.1038/s41438-020-0291-7)

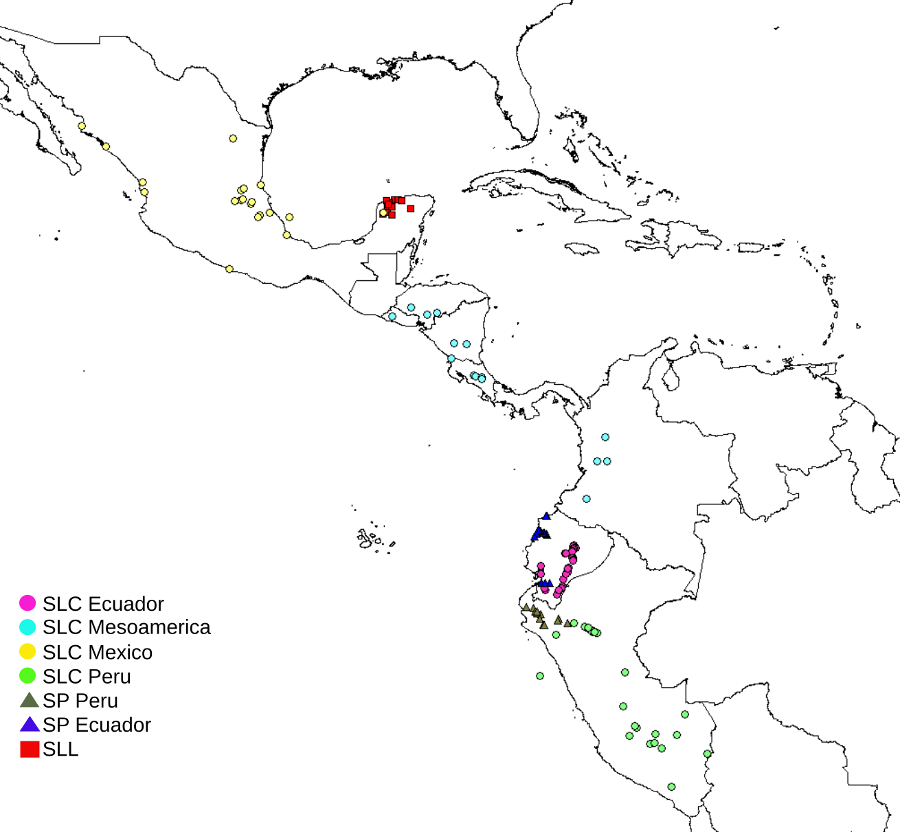

Supplement: Supplementary file 1 — Figure S1 [file 41438_2020_291_MOESM1_ESM.png]

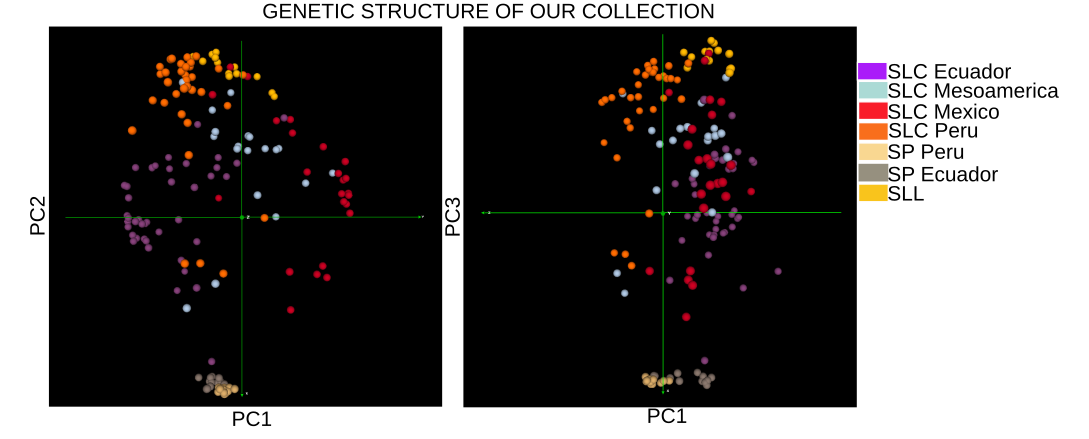

Supplement: Supplementary file 2 — Figure S3 [file 41438_2020_291_MOESM2_ESM.png]

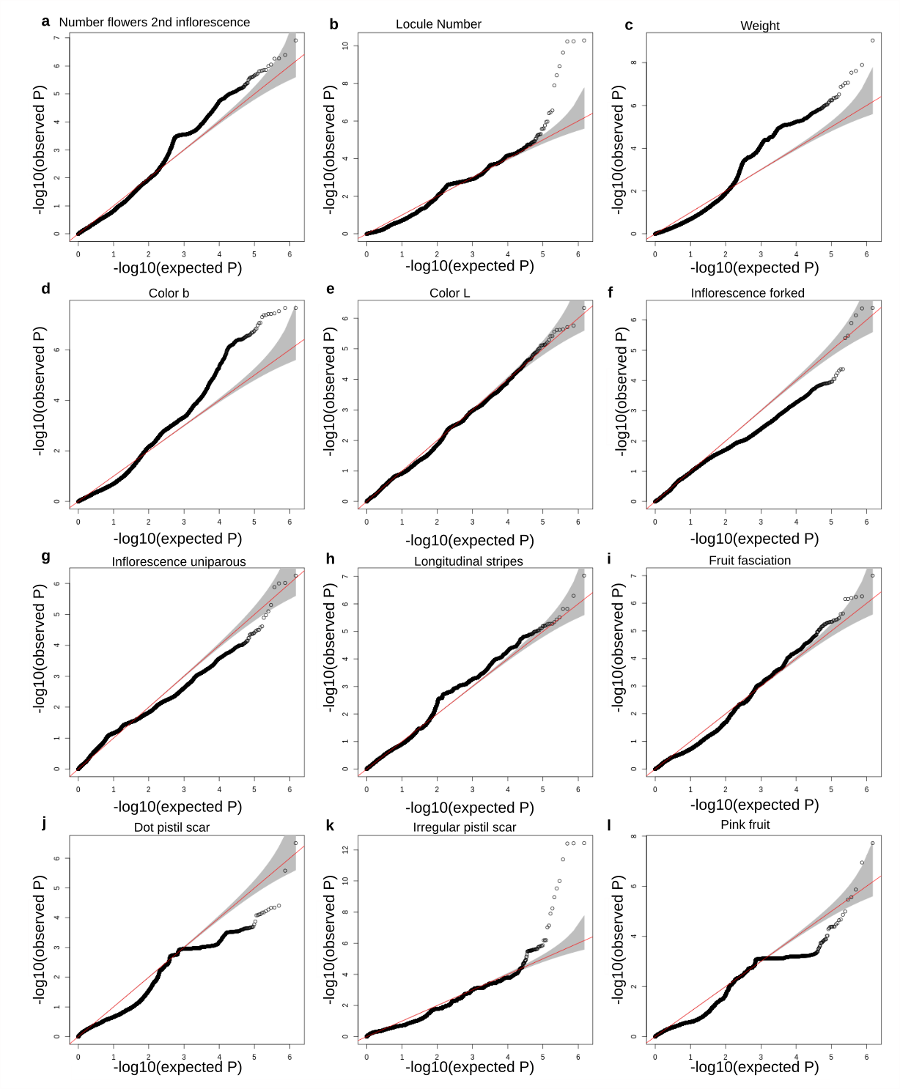

Supplement: Supplementary file 3 — Figure S4 [file 41438_2020_291_MOESM3_ESM.png]

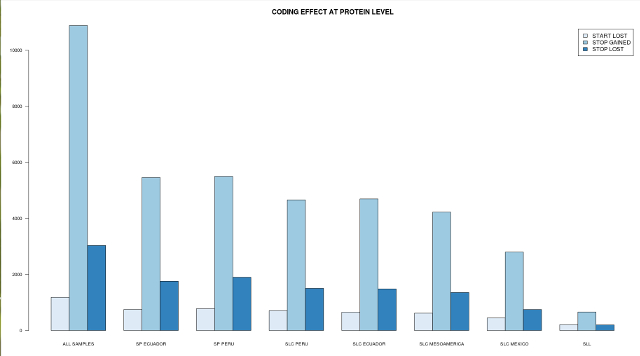

Supplement: Supplementary file 5 — Figure S2 [file 41438_2020_291_MOESM5_ESM.jpg]
