# Supplementary material for: Exploiting the diversity of tomato: the development of a phenotypically and genetically detailed germplasm collection
Source: Hortic Res. 2020 May 1;7:66. doi: 10.1038/s41438-020-0291-7 (PMC7192925; doi:10.1038/s41438-020-0291-7)
Supplement: Supplementary file 6 — Table S2 [file 41438_2020_291_MOESM6_ESM.pdf]

name	description	ontology_id	ontology_name	ontology_description
length from 1st to 10th leaf	The distance between the 1st and the 10th leaf in cm. Recorded at complete developmental stage.	SP:0000428	stem length between 1st and 10th true leaves (cm)	The distance between the 1st and the 10th leaf in cm. Recorded at complete developmental stage.
number of leaves between 2nd and 3rd inflorescence	number of leaves between 2nd and 3rd inflorescence. Recorded at complete developmental stage.	SP:0000399	number of leaves between 2nd and 3rd inflorescence	number of leaves between 2nd and 3rd inflorescence
number of leaves between 3rd and 4th inflorescence	number of leaves between 3rd and 4th inflorescence. Recorded at complete developmental stage.	SP:0000400	number of leaves between 3rd and 4th inflorescence	number of leaves between 3rd and 4th inflorescence
height until first inflorescence (cm)	Plant height until first inflorescence. Recorded at complete developmental stage.	SP:0000403	plant height until first inflorescence	The height of the plant until the first inflorescence in cm
height until last inflorescence (cm)	Plant height until last inflorescence. Recorded at complete developmental stage.	SP:0000404	plant height until last inflorescence	The height of the plant until the last inflorescence in cm
total number of inflorescences	Total number of inflorescence. Recorded at complete developmental stage.	SP:0000405	total number of inflorescences	Count of the number of inflorescences.

Stem width between 2nd and 3rd inflorescence (mm)	Stem width between 2nd and 3rd inflorescence, measured with vernier caliper. Recorded at complete developmental stage.	SP:0000407	Stem width between 2nd and 3rd inflorescence (mm)	Stem width between 2nd and 3rd inflorescence, measured in mm at complete developmental stage.
Stem width between 3rd and 4th inflorescence (mm)	Stem width between 3rd and 4th inflorescence, measured with vernier caliper. Recorded at complete developmental stage.	SP:0000408	Stem width between 3rd and 4th inflorescence (mm)	Stem width between 3rd and 4th inflorescence, measured in mm at complete developmental stage.
inflorescence lenght 2	Length measured in the second inflorescence.	SP:0000429	inflorescence length	
inflorescence lenght 3	Length measured in the third inflorescence.	SP:0000429	inflorescence length	The distance from the stem to the last flower along the rachis of the inflorescence.
Number flowers in 2nd inflorescence	The number of flowers in the second inflorescence.	SP:0000409	number of flowers in second inflorescence	Count the number of flowers in the second inflorescence.
number flowers in 3rd inflorescence	The number of flowers in the third inflorescence.	SP:0000410	number of flowers in third inflorescence	Count the number of flowers in the third inflorescence.
Number of petals	Average of the number of petals in three flowers, randomly selected, per plant.	SP:0000414	number of petals	Count petals per flower.

Number sepals	Average of the number of sepals in three flowers, randomly selected, per plant.	SP:0000413	number of sepals	Count sepals per flower.
Petal length	Average length of petals from a representative flower per plant. Length measured in cm. from the centre of the flower to the end of the petal.	SP:0000415	petal lengh	Average length of petals from a representative flower per plant. Length measured in cm. from the centre of the flower to the end of the petal. Comment
Petal width	Average width of petals from a representative flower per plant. Width measured in cm. between the join point of the petals.	SP:0000416	petal width	Average width of petals from a representative flower per plant. Width measured in cm. between the join point of the petals.
Sepal length	Average length of sepals from a representative flower per plant. Length measured in cm. from the pedicel to the end of the sepal.	SP:0000417	sepal lengh	Average length of sepals from a representative flower per plant. Length is measured in cm. from the pedicel to the end of the sepal.

Sepal width	Average width of sepals from a representative flower per plant. Width measured in cm. between the join point of the sepals.	SP:0000418	sepal width	Average width of sepals from a representative flower per plant. Width was measured in cm. between the join point of the sepals.
color L	Four mature fruits is the maximum number of fruits per plant used to measure color: Data was recorded in a point close to pistil scar and color is recorded in L*a*b color system. Mean L, Mean a and Mean b are the average of the four measures/plant: for example, mean L(average between L1, L2, L3 and L4).	SP:0000433	epicarp Lab-scale 'L' value	Epicarp 'L' color value was recorded in a point close to pistil scar. Color is recorded in L*a*b color system using a colorimeter.
color a	Four mature fruits is the maximum number of fruits per plant used to measure color: Data was recorded in a point close to pistil scar and color is recorded in L*a*b color system. Mean L, Mean a and Mean b are the average of the four measures/plant: for example, mean L(average between L1, L2, L3 and L4).	SP:0000434	epicarp Lab-scale 'a' value	Epicarp 'a' color value was recorded in a point close to pistil scar. Color is recorded in L*a*b color system using a colorimeter.

color b	Four mature fruits is the maximum number of fruits per plant used to measure color: Data was recorded in a point close to pistil scar and color is recorded in L*a*b color system. Mean L, Mean a and Mean b are the average of the four measures/plant: for example, mean L(average between L1, L2, L3 and L4).	SP:0000435	epicarp Lab-scale 'b' value	Epicarp 'b' color value was recorded in a point close to pistil scar. Color is recorded in L*a*b color system using a colorimeter.
Fruit weight	the average between the weight and the number of fruits (8 fruits is the maximum number of fruits weigthed per accession)	SP:0000080	fruit mass, weight in grams	The mass of the fruit measured in grams.
Number of primary leaflets	Recorded in leaves that were fully opened when the plant was completely developed. We measure 2 leaves per plant.	SP:0000437	number of primary leaflets per leaf	Number of primary leaflets per leaf. Counting in fully opened leaves when the plant is completely developed.
Number of small leaflets	Recorded in leaves that were fully opened when the plant was completely developed. We measure 2 leaves per plant.	SP:0000438	number of secondary leaflets per leaf	Number of secondary leaflets per leaf. Counting in fully opened leaves when the plant is completely developed.

Leaf lenght	Recorded in leaves that were fully opened when the plant was completely developed. We measure the length from the apex to the basal zone in 2 leaves per plant.	SP:0000229	leaf length	leaf length from the apex to the basal zone in cm.
Leaf width	Recorded in leaves that were fully opened when the plant was completely developed. We measure the distance between the 2 largest primary leaflets in 2 leaves per plant.	SP:0000041	leaf width	Leaf width in cm.
Petals Curvature	1-3 score (3:low, 5:intermediate, 7:strong). The common position of the petals from fully opened flowers.	SP:0000442	petals curvature observation 1-7 scale	The common position of the petals of fully opened flowers. 1-7 score (3:low, 5:intermediate, 7:strong).
Stamen colour	3-7 score (3:yellow, 5:pale orange, 7:orange). Stamen color from fully opened flowers, randomly selected.	SP:0000444	stamen color observation 3-7 scale	Stamen color of fully opened flowers. 3-7 scale (3:yellow, 5:pale orange, 7:orange).
Colour immature fruit	3-9 score (3:green-white, 5:ligth green, 7:green, 9:dark green). Fruit colour recorded before maturity. Different fruits were observed in whole plant.	SP:0000448	immature green fruit epicarp color, observation nominal scale 3-9	Color observation of immature green fruit. 3-9 score (3:green-white, 5:light green, 7:green, 9:dark green).

Exterior colour of mature fruit	0:6 score (0:yellow, 1:yellow-orange, 2:orange, 3:red-orange, 4:pink, 5:red, 6:intense red)	SP:0000449	epicarp fruit color, observation nominal scale 0-6	Fruit color of the epicarp. Exterior color of mature fruit. 0-6 score (0:yellow, 1:yellow-orange, 2:orange, 3:red-orange, 4:pink, 5:red, 6:intense red).
Skin color ripe	0-1 score (0:colourless, 1:yellow). The colour of the peeled fruit skin of a mature fruit.	SP:0000450	e fruit skin color. Nominal score 0-1	The color of the fruit epidermis. 0-1 score (0:colorless, 1:yellow).
Width pedicel scar	3-7 score (3:narrow, 5:medium, 7:wide). Recorded at the widest part of pedicel scar in randomly fruits of the whole plant.	SP:0000452	fruit pedicel scar width. Observation 3-7 nominal scale	Scar width at the widest part of the pedicel scar. 3-7 score (3:narrow, 5:medium, 7:wide).
Ribbing calyx end	0-9 score (0:absent, 3:very weak, 5:weak, 7:intermediate, 9:strong)	SP:0000461	Ribbing calyx end. Observation 0-9 scale	An arrangement of ribs surrounding the calyx end of a full size fruit. 0-9 score (0:absent, 3:very weak, 5:weak, 7:intermediate, 9:strong).

Vascular Content	0-7 score (0:absent, 3:sligth, 5:intermediate, 7:severe)	SP:0000465	vascular bundle content	Scored in mature green fruit. Vascular bundles network for water transportation. It begins in the stem scar and runs to the locules through the placenta while also extending to the outer pericarp. Visual score 0-7 (0:absent, 3:slight, 5:intermediate, 7:severe).
Longitudinal stripes	0-1 score (0:absence, 1:presence). Presence of longitudinal stripes in immature fruits.	SP:0000454	longitudinal stripes presence on immature green fruits. Observation 0-1 scale	Presence of longitudinal stripes on immature fruits. 0-1 score (0:absence, 1:present).
Style exertion	1-4 score (1:inserted, 2:same level as stamen, 3:slightly exerted, 4:highly exerted).)The position of the style compared with stamens. Different flowers of the plant were observed.	SP:0000446	style exertion. Visual rating 1-4	

Leaf Dissection	0-2 score (0:low, 1:intermediate, 2:high) This measure summarizes the extent of dissection in a leave. Recorded in leaves that were fully opened when the plant was completely developed. We measure 2 leaves per plant.	SP:0000456	leaf dissection. Observation 0-2 nominal scale	The extent of dissection in a fully opened leaf. 0-2 score (0:low, 1:intermediate, 2:high).
Leaf border	0-3 score (0:entire, 1:undulating, 2:serrated, 3:strong serrated). This measure describes the outline of the leave. Recorded in leaves that were fully opened when the plant was completely developed. We measure 2 leaves per plant.	SP:0000458	leaf border variable. Observation 0-3 nominal scale	The outline of fully opened leaf. 0-3 score (0:entire, 1:undulate, 2:serrated, 3:strong serrated).
Leaf attitude	1-9 score to describe the way that leaves are held naturally (1:semi-erect, 3:semi-horizontal, 5:horizontal, 7:horizontal-drooping, 9:drooping). Recorded at complete developmental stage of the plant. The plant was observed in general to take the data.	SP:0000440	leaf habit visual rating 1-9 scale	Description of the way the leaves are held naturally. Recorded at complete developmental stage of the plant. Visual rating 1-9 score (1:semi-erect, 3:semi-horizontal, 5:horizontal, 7:horizontal-drooping, 9:drooping).

Antho Stem	Anthocyanin content was measure in stem from adult plants.	SP:0000441	stem anthocyanin content	Anthocyanin content in the stem of mature plants.
Stamen colour	3-7 score (3:yellow, 5:pale orange, 7:orange). Stamen color from fully opened flowers.	SP:0000444	stamen color observation 3-7 scale	Stamen color of fully opened flowers. 3-7 scale (3:yellow, 5:pale orange, 7:orange).
Leaf type	0-2 score (0:pimpinellifolium, 1:standard, 2:double feathered)	SP:0000467	leaf type. Observation	0-2 score (0:pimpinellifolium, 1:standard, 2:double feathered).
Growth habit	0-2 score (0:Determinate, 1:Semi-determinate, 2:Indeterminate)	SP:0000470	plant growth habit, visual observation nominal scale	plant growth habit, visual observation nominal scale
Leaf Colour	3-7 score (3:Light green, 5:medium green, 7:dark green)	SP:0000472	green leaf color, visual observation 3-7 scale	3:Light green, 5:medium green, 7:dark green.
inflorescence fishbone type	0-1 score (0:absence, 1:presence). This trait was observed in the 2nd and 3rd inflorescence. The types were recorded separately, because 2nd and 3rd inflorescence could be different.	SP:0000474	inflorescence type, nominal scale	1: uniparous, 2: fishbone, 3: forked, 4-irregular.

inflorescence forked type	0-1 score (0:absence, 1:presence). This trait was observed in the 2nd and 3rd inflorescence. The types were recorded separately, because 2nd and 3rd inflorescence could be different.	SP:0000474	inflorescence type, nominal scale	1: uniparous, 2: fishbone, 3: forked, 4-irregular.
inflorescence irregular type	0-1 score (0:absence, 1:presence). This trait was observed in the 2nd and 3rd inflorescence. The types were recorded separately, because 2nd and 3rd inflorescence could be different.	SP:0000474	inflorescence type, nominal scale	1: uniparous, 2: fishbone, 3: forked, 4-irregular.
inflorescence uniparous type	0-1 score (0:absence, 1:presence). This trait was observed in the 2nd and 3rd inflorescence. The types were recorded separately, because 2nd and 3rd inflorescence could be different.	SP:0000474	inflorescence type, nominal scale	1: uniparous, 2: fishbone, 3: forked, 4-irregular.
Leafy inflorescence	0-2 score (0:absence, 1:leaves, 2:leaves and shoots)	SP:0000475	leafy inflorescence , observation 0-2 scale	Scored when the inflorescence is completely developed. 0-2 score (0:absent, 1:leaves, 2:leaves and shoots).

Petal colour	3-7 score (3:Pale yellow, 5:Yellow, 7:Yellow-orange)	SP:0000477	flower color, observation 1-7 nominal scale	3: Pale yellow, 5: Yellow, 7: Yellow-orange.
Green shoulder	0-7 score (0:Uniform, 3:Light green, 5: Medium green, 7:Dark green)	SP:0000478	proximal fruit end color, observation 0-7 nominal scale	0: Uniform, 3: Light green, 5: Medium green, 7: Dark green.
Shape pistil scar	1-7 score (1:dot, 3:linear, 5:estrellate, 7:irregular)	SP:0000480	distal fruit end scar shape, observation 1-7 nominal scale	1: dot, 3: linear, 5: star, 7: irregular.
Fruit fasciation	0-7 score (0-Not present, 3-Low, 5-Intermediate, 7-Severe)	SP:0000482	fruit fasciation, observation 0-7 ordinal scale	0: Not present, 3: low, 5: intermediate, 7: severe.
Locule number	locules in the transversal sect	SP:0000089	cule number, co	ocules in the fruit. This data is taken in the tran

[illegible]sversal section.
